# Education as a predictor of liver testing behaviour: Insights from a large-scale MASLD awareness survey in India

**DOI:** 10.1371/journal.pone.0335857

**Published:** 2025-11-21

**Authors:** Kanica Kaushal, Manoj Kumar Sharma, Priyanka Aggarwal, Smriti Singh, Sumridhi Gautam, Anamika Tomar, Guresh Kumar, Siddhesh Mhatre

**Affiliations:** 1 Department of Clinical Research and Epidemiology, Institute of Liver and Biliary Sciences, New Delhi, India; 2 Department of Hepatology, Institute of Liver and Biliary Sciences, New Delhi, India; 3 Department of Community Medicine, Rajasthan University of Health Sciences (RUHS) College of Medical Sciences, Jaipur, Rajasthan, India; 4 Consultant Statistics, Institute of Liver and Biliary Sciences, New Delhi, India; Niigata University: Niigata Daigaku, JAPAN

## Abstract

**Introduction:**

Metabolic dysfunction-associated steatotic liver disease (MASLD) is a growing public health concern, yet awareness remains low. This study aimed to assess MASLD awareness and identify predictors of liver testing behavior in Jaipur, India.

**Methods:**

A cross-sectional survey of 2,102 adults was conducted from October 2023 to March 2024. Participants completed a questionnaire assessing liver health knowledge, awareness, and testing history. Logistic regression analyzed predictors of liver testing.

**Results:**

Only 5.9% of participants had heard of fatty liver disease, with 94.0% completely unaware. Knowledge of liver functions was extremely low, with only 14.9% recognizing food digestion as a liver function. The liver testing rate was 1.8% overall. Education emerged as the strongest predictor of testing behavior, with graduates and above 8.35 times more likely to be tested than non-graduates (OR=8.35, 95% CI: 3.80–18.37, p < 0.001). Participants with higher liver function knowledge had dramatically higher testing rates of 12.9% versus 1.2% for those with low knowledge (OR=9.35, 95% CI: 4.2–20.8, p < 0.001). Employment status also significantly predicted testing (OR=5.42, 95% CI: 2.8–10.5, p < 0.001).

**Discussion:**

This study reveals a catastrophic knowledge deficit regarding MASLD, with 94% of participants completely unaware of the condition. The strong association between education, knowledge, and testing behavior highlights a critical knowledge-to-action pathway. The extremely low overall testing rate (1.8%) indicates massive underutilization of liver health screening, particularly among unemployed and less educated individuals. These findings underscore the urgent need for comprehensive public health education on liver health, especially given India’s rising MASLD prevalence and young demographic profile. Targeted interventions to improve awareness and facilitate testing access among socioeconomically disadvantaged groups are essential to address this public health crisis.

## Introduction

Fatty liver disease, encompassing both non-alcoholic fatty liver disease (NAFLD) and alcohol-associated liver disease (ALD), is rapidly emerging as a significant public health concern worldwide [1]. Its increasing prevalence is strongly linked to rising rates of obesity, type 2 diabetes mellitus, and sedentary lifestyles—factors that have become more pronounced in developing countries, including India [2] In recent years, the terminology surrounding NAFLD has evolved, with international consensus groups recommending the adoption of the terms metabolic dysfunction-associated fatty liver disease (MAFLD) and, more recently, metabolic dysfunction-associated steatotic liver disease (MASLD). Despite this shift, NAFLD remains widely used in clinical and public health contexts in India, including during the design and implementation of this survey. We retained the term MASLD throughout this manuscript for consistency, while acknowledging this evolving nomenclature to enhance the relevance of our findings to a global audience. MASLD, estimated to affect approximately 25% of the world’s adult population, is the leading cause of chronic liver disease globally [1,3,4].

MASLD encompasses a range of conditions, from simple steatosis to steatohepatitis (MASH), fibrosis, and cirrhosis. Up to 1/3 of NAFLD patients will have NASH which is a risk factor for fibrosis progression, and approximately 40% of NASH patients will experience fibrosis progression. The recent estimated annual progression of fibrosis from NAFLD is up to 0.09% with an incidence of advanced fibrosis as 70 per 1000 patients [5,6]. Hepatocellular carcinoma and liver cirrhosis present significant global health challenges, primarily due to chronic viral infections such as hepatitis B (HBV) and C (HCV), as well as MASLD [7,8]. Previously, MASLD was considered a condition primarily affecting wealthier nations. However, recent research shows that it is now just as common in developing countries like India [9]. As the prevalence of non-communicable diseases such as diabetes, hypertension, obesity, and dyslipidemia continues to rise, MASLD is reaching epidemic proportions [10]. It has now become the leading cause of liver disease globally.

MASLD is considered the hepatic component of the metabolic syndrome (MetS) and is recognized as part of a multi-system disease. MASLD prevalence is higher in patients with type 2 diabetes mellitus (T2DM) than in the general population, while the incidence of T2DM is higher in patients with MASLD [11]. Metabolic syndrome (MetS) is a prevalent obesity-related condition. As the name suggests, it causes metabolic dysfunction in various body tissues, including the liver. This dysfunction often leads to fat accumulation in the liver, contributing to the development of fatty liver disease [12].

Despite its widespread occurrence and potential to progress to advanced liver fibrosis, cirrhosis, and hepatocellular carcinoma, awareness about fatty liver disease among the public remains inadequate [13]. This is particularly concerning in India, where urbanization and lifestyle transitions are contributing to an increased incidence of metabolic dysfunction–associated liver conditions, even among younger age groups [14]. Liver diseases continue to pose a significant health concern despite ongoing efforts to manage them. Early detection and prevention of fatty liver disease rely heavily on public awareness, lifestyle modification, and timely medical consultation. However, studies have shown that the knowledge of risk factors, symptoms, and complications of fatty liver disease among the general population is limited [15]. In this context, health education campaigns can serve as vital tools in raising awareness and encouraging healthy behaviours. Structured educational interventions have been shown to significantly improve knowledge and self-efficacy in managing non-communicable diseases [16].

This study aims to assess the public’s awareness and understanding of MASLD and to identify gaps in current public health education campaigns. Few surveys have explored public knowledge of MASLD within Asian communities. The findings will offer valuable insights into the unmet needs in public education about liver health, supporting efforts to enhance awareness and understanding of liver-related issues among the general population.

## Methodology

This cross-sectional survey was conducted in Jaipur, Rajasthan, India, from October 2023 to March 2024. The study employed a quantitative approach using structured questionnaires to assess basic knowledge regarding MASLD among the general adult population. The research evaluated public awareness levels and identified educational gaps that could inform targeted health promotion strategies. The study included adults aged 18 years and older who were residents of Jaipur, Rajasthan, who could understand and respond to questions in Hindi or English and provided voluntary informed consent to participate. Individuals under 18 years of age, those with cognitive impairment preventing informed consent, participants unable to complete the survey due to language barriers, and those with incomplete survey responses were excluded from the study. A convenience sampling approach was employed to recruit 2,102 participants across Jaipur’s urban, semi-urban, and rural areas. A convenience sampling approach was employed to recruit 2,102 participants across Jaipur, Rajasthan, from October 2023 to March 2024. Trained surveyors recruited participants through multiple channels, including primary and community health centers. The sample included participants from urban (60.0%, n = 1,261), semi-urban (25.0%, n = 525), and rural (15.0%, n = 316) areas. Potential participants were screened for eligibility (adults ≥18 years, Jaipur residents, ability to understand Hindi/English, and provision of informed consent). The sample size was calculated to adequately represent the general population with sufficient power to detect meaningful differences in awareness levels across demographic subgroups. Participants were recruited through multiple channels, including healthcare facilities, to ensure demographic diversity. A questionnaire with 24 questions on liver health awareness was developed in English and Hindi—the first section’s 11 questions covered sociodemographic data, including personal details and medical history. The second section had five questions on liver function knowledge with “Yes/No/Don’t know” responses. The third section had 1 question on liver disease awareness. The fourth section assessed liver protection knowledge through 8 questions with “Agree/Disagree/Not sure” options. The final section contained 10 questions on fatty liver disease covering symptoms, complications, and diagnostics. High knowledge was defined as correctly answering at least two out of five liver function questions. This threshold was selected because the distribution of scores showed that most participants answered only one or none correctly. In contrast, a small subgroup with ≥2 correct answers exhibited distinctly higher testing rates (12.9% vs. 1.2%).

“Graduate and above” status was defined as individuals who had completed an undergraduate or postgraduate degree. All other education levels—including primary school, secondary school (8th grade), high school (10th grade), and intermediate (12th grade)—were categorized as “non-graduate.” This classification means that the “non-graduate” group included individuals with education ranging from only primary schooling up to, but not including, a full undergraduate degree. In the Indian context, under the Right to Education Act (2009), free and compulsory education is provided up to Grade 8 (approximately age 14). Therefore, the non-graduate category encompasses both those who completed only compulsory schooling and those who pursued additional years of education but did not attain a degree. This context is essential for interpreting the association between education level, health literacy, and testing behaviour in our findings.

Logistic regression was used to analyse relationships between demographic factors, knowledge scores, and liver testing behaviour. Two regression approaches were used: logistic regression estimated odds ratios for categorical predictors of liver testing, while linear regression examined the continuous association of knowledge, attitude, and practice scores with liver testing likelihood. The logistic model provides relative risk interpretation, whereas the linear model highlights the strength and direction of continuous predictors. Due to the small number of testing events, ROC analysis was not performed, as it would not yield stable estimates.

Survey responses were entered into Microsoft Excel and transferred to STATA 17 and SPSS 23 for statistical analysis. Data cleaning included checking for missing values, outliers, and inconsistencies. Variables were coded appropriately, and continuous variables were assessed for normality. A scoring system was developed with 1 point for correct responses and 0 for incorrect ones. Total knowledge scores were calculated by summing correct responses across domains, while domain-specific scores were computed for liver function knowledge, disease awareness, risk factors, and prevention understanding. Descriptive analysis presented continuous variables as mean ± standard deviation and categorical variables as frequencies and percentages, with age group stratification and demographic cross-tabulations. Inferential statistics included logistic regression to identify predictors of liver testing behaviour, Chi-square tests for demographic associations, odds ratios with 95% confidence intervals, and multivariate analysis for confounding variables.

The study protocol and questionnaire received institutional ethical approval. All participants provided informed verbal consent after receiving detailed information about the study’s purpose, procedures, confidentiality measures, and their right to withdraw. No personal identifying information was collected beyond what was necessary for demographic analysis, ensuring participant anonymity.

## Results

Of approximately 2,350 individuals approached, 2,280 met eligibility criteria, and 2,102 agreed to participate (response rate: 92.2%). Primary reasons for refusal included time constraints and lack of interest. Surveys were administered in either Hindi (65.0%) or English (35.0%), based on participant preference. A total of 2,102 participants completed the survey over months, from October 2023 to March 2024. The mean age of participants was 35.08 ± 13.31 years, with female predominance (55.3%, n = 1,162) compared to males (44.7%, n = 940). Employment status revealed concerning patterns with 63.8% (n = 1,341) unemployed, 22.3% (n = 468) self-employed, and only 13.9% (n = 293) in government or private service jobs. Educational attainment showed 27.4% (n = 576) were undergraduates, 16.3% (n = 343) had no formal education, and 3.7% (n = 78) were postgraduates. Despite about one-third of participants having graduate-level education (31.1%), unemployment remained high, suggesting potential economic challenges or job market barriers.

In India, students are often categorized as unemployed since they remain financially dependent on their parents during their education. Consequently, the ‘unemployed’ group in our dataset likely includes a substantial number of younger individuals, which may partly explain the lower liver testing rates observed in this category. This potential age-related confounding has been acknowledged as a limitation in the discussion.

Table 1 shows that 81.8% of the population was under 45 years of age, representing a predominantly young demographic at early risk of developing lifestyle-related conditions. The data also indicate high unemployment levels despite a relatively well-educated population (around one-third graduates and above), suggesting possible underemployment or economic barriers rather than a true employment–education paradox. Additionally, the region’s unique linguistic and geographic diversity may influence community health and socioeconomic patterns. Analysis of liver function knowledge revealed significant deficits across all physiological domains. Food digestion was the most recognized liver function, correctly identified by 14.9% (n = 313) participants. Other functions showed minimal recognition: blood filtration by 3.7% (n = 77), vitamin storage by 2.4% (n = 50), blood clotting by 1.8% (n = 37), and energy storage by only 1.1% (n = 23). Most participants responded ‘Don’t know’ to liver function questions, indicating widespread ignorance about basic liver physiology.

**Table 1:** Socio-demographic characteristics of study participants (N = 2,102).

Characteristic	Categories	n (%)
**Age (years)**	Mean ± SD	35.08 ± 13.31 years
	18-30	1,035 (49.2)
	31-45	685 (32.6)
	46-60	255 (12.1)
	>60	125 (5.9)
**Gender**	Female	1,162 (55.3)
	Male	940 (44.7)
**Employment Status**	Students	301 ()
	Unemployed	1,040 (63.8)
	Self-employed	468 (22.3)
	Service (Govt/Private)	291 (13.9)
**Education Level**	Undergraduate	576 (27.4)
	None	343 (16.3)
	High School (10th)	331 (15.7)
	Intermediate (12th)	324 (15.4)
	Secondary School (8th)	251 (11.9)
	Primary School (5th)	199 (9.5)
	Postgraduate	78 (3.7)
**Geographic Distribution**	Urban Jaipur	1,261 (60.0)
	Semi-urban areas	525 (25.0)
	Rural areas	316 (15.0)
**Language of Survey**	Hindi	1,366 (65.0)
	English	736 (35.0)
**Medical Conditions**	None reported	1,941 (92.3)
	Has existing conditions	161 (7.7)

Table 2 highlights that 84.7% of individuals can’t recall their last checkup, presents the prevalence of diabetes and hypertension—conditions known to increase MASLD risk—and notes that 51.1% are overweight or obese, raising disease risk.

**Table 2:** Health behaviour and medical history (N = 2,102).

Characteristic	Categories	n (%)
**Last Health Check-up**	Cannot recall	1,780 (84.7)
	Within 6 months	168 (8.0)
	6-12 months ago	105 (5.0)
	More than 1 year ago	49 (2.3)
**History of Diabetes**	No	1,891 (90.0)
	Yes	211 (10.0)
**History of Hypertension**	No	1,786 (85.0)
	Yes	316 (15.0)
**Family History of Liver Disease**	No/Unknown	1,997 (95.0)
	Yes	105 (5.0)
**Regular Exercise**	No	1,471 (70.0)
	Yes	631 (30.0)
**Alcohol Consumption**	No	1,681 (80.0)
	Occasional	315 (15.0)
	Regular	106 (5.0)
BMI Categories*	Normal (18.5–22.9)	924 (44.0)
	Overweight (23–24.9)	756 (36.0)
	Obese (≥25)	317 (15.1)
	Underweight (<18.5)	105 (5.0)

Table 3 shows that the MASLD awareness assessment highlights a significant catastrophic knowledge deficit. Notably, 94.0% of respondents were uncertain rather than outright disagreeing, and 97.2% were unaware that MASLD is preventable, indicating a critical therapeutic gap. Awareness regarding fatty liver disease was catastrophically low, with 94% of participants completely unaware of the condition. This profound lack of knowledge was reflected in behaviour, as only 1.8% of respondents had ever undergone a liver test—demonstrating severe underutilization of preventive health screening.

**Table 3:** MASLD awareness and knowledge assessment (N = 2,102).

Assessment Domain	Categories	n (%)
**Heard of “Fatty Liver”**	Not sure	1,976 (94.0)
	Yes	123 (5.9)
	No	3 (0.1)
**Fatty Liver is Preventable**	Not sure/Don’t know	2,043 (97.2)
	Yes	59 (2.8)
**Health Check-up Includes Liver Tests**	Not sure/Don’t know	2,068 (98.4)
	Yes	34 (1.6)
**Non-alcoholic Beverages Can Cause Fatty Liver**	Not sure/Don’t know	2,078 (98.9)
	Yes	24 (1.1)
**Fatty Liver Can Be Cured in Early Stages**	Not sure/Don’t know	2,066 (98.3)
	Yes	36 (1.7)
**Fat in Liver Can Cause Serious Health Problems**	Not sure/Don’t know	2,062 (98.1)
	Yes	40 (1.9)

Table 4 shows disease recognition and risk factor awareness with multiple responses allowed per domain, revealing severe knowledge deficits across all liver health areas. The analysis demonstrates that 90.1% of participants could not recognize any liver-associated diseases, with only 9.9% able to identify at least one condition. Among specific diseases, cancer had the highest recognition (9.4%), followed by hepatitis (8.4%), fatty liver (5.8%), and cirrhosis (4.0%). Risk factor awareness was even more concerning, with 95.0% of participants unable to identify any fatty liver risk factors. Notably, only 5.0% recognized the role of excessive alcohol in liver disease, while recognition of other key risk factors was extremely low: obesity (3.0%), diabetes (2.0%), and poor diet (1.5%). Protective measure awareness showed the highest knowledge levels, with 24.9% of participants recognizing at least one protective strategy and 75.1% demonstrating no knowledge of prevention methods. Regular exercise (8.9%) and balanced diet (9.4%) were the most recognized protective measures, while awareness of weight management (4.0%), regular check-ups (2.3%), and limited alcohol consumption (3.0%) remained low. These findings illustrate how insufficient awareness across all domains—disease recognition, risk factors, and protective measures—contributes to poor health-seeking behaviours and the extremely low 1.8% liver testing rate observed in this population.

**Table 4:** Disease recognition and risk factor awareness (N = 2,102).

Domain	Specific Item	Correctly Identified n (%)
**Liver-Associated Diseases**	Cancer	197 (9.4)
	Hepatitis	176 (8.4)
	Fatty liver	122 (5.8)
	Cirrhosis	84 (4.0)
	**At least one disease recognized**	**208 (9.9)**
	**No diseases recognized**	**1,894 (90.1)**
**Fatty Liver Risk Factors**	Excessive alcohol	105 (5.0)
	Obesity	63 (3.0)
	Diabetes	42 (2.0)
	Poor diet	31 (1.5)
	**At least one risk factor known**	**106 (5.0)**
	**No risk factors known**	**1,996 (95.0)**
**Protective Measures**	Regular exercise	187 (8.9)
	Balanced diet	197 (9.4)
	Weight management	84 (4.0)
	Regular check-ups	48 (2.3)
	Limited alcohol	63 (3.0)
	**At least one measure known**	**523 (24.9)**
	**No measures known**	**1,579 (75.1)**

*Multiple responses were allowed.

Table 5 logistic regression analysis revealed education level as the strongest predictor of liver testing behaviour, with graduates and above having 8.35-fold higher odds of undergoing liver testing compared to non-graduates (OR = 8.35, 95% CI: 3.80–18.37, p < 0.001), corresponding to testing rates of 4.5% versus 0.5%, respectively. Employment status emerged as the second strongest predictor, with employed individuals showing significantly higher testing rates (6.1%) compared to unemployed participants (1.2%) (OR = 5.42, 95% CI: 2.8–10.5, *p* < 0.001), suggesting workplace health benefits may facilitate access to liver screening. Knowledge score demonstrated a strong knowledge-to-action pathway, with participants having higher liver function knowledge (≥ 2 correct answers) exhibiting markedly higher testing rates (12.9%) versus 1.2% for those with low knowledge (OR = 9.35, 95% CI: 4.2–20.8, *p* < 0.001), underscoring the importance of health education in promoting preventive behaviours. Gender differences were modest but significant, with males twice as likely as females to have undergone liver testing (OR = 2.06, 95% CI: 1.1–3.9, *p* < 0.05), corresponding to testing rates of 2.4% versus 1.2%, possibly reflecting occupational screening requirements or differences in health-seeking behaviour.

**Table 5:** Predictors of liver testing behaviour.

Predictor Variable	Category	Tested/Total	Rate (%)	Odds Ratio	95% CI	Statistical Significance
**Overall Rate**	All participants	37/2,102	1.8%	–	–	–
**Education Level**	Graduate and above	29/654	4.5%	8.35	3.80 −18.37	p < 0.001
	Non-graduate	8/1,448	0.5%	Reference	–	–
**Employment Status**	Employed (Govt/Private)	18/293	6.1%	5.42	2.8-10.5	p < 0.001
	Unemployed	16/1,341	1.2%	Reference	–	–
**Gender**	Male	23/940	2.4%	2.06	1.1-3.9	p < 0.05
	Female	14/1,162	1.2%	Reference	–	–
Knowledge Score*	High knowledge (≥2)	12/93	12.9%	9.35	4.2-20.8	p < 0.001
	Low knowledge (<2)	25/2,009	1.2%	Reference	–	–

*High knowledge is defined as correctly answering ≥2 liver function questions.

Table 6 linear regression analysis further reinforced these findings by identifying knowledge score as the only statistically significant predictor of liver testing behaviour (p = 0.0126). Participants with higher liver health knowledge were nearly 49 times more likely to undergo testing (OR = 48.7, 95% CI: 2.2–1076.4). However, the vast confidence interval reflects instability in this estimate due to the small number of testing events (n = 37). Attitude and practice scores were not significant predictors (p > 0.99), suggesting that specific liver health knowledge, rather than general health attitudes, drives testing behaviour. These findings validate the critical knowledge-to-action pathway and support targeted educational interventions as the most effective strategy for improving liver screening uptake in this population.

**Table 6:** Predictors of liver testing behaviour.

Predictor	Estimate (β)	Std. Error	z-value	p-value
Intercept	210	1.38 × 10^6^	0	0.9999
Knowledge Score	3.89	1.561 × 10^0^	2.494	0.0126*
Attitude Score	−140.7	4.60 × 10^5^	0	0.9998
Practice Score	138.4	2.35 × 10^4^	0.006	0.9953

## Discussion

This study reveals a catastrophic knowledge deficit regarding MASLD among the general population in Jaipur, Rajasthan. Our analysis of 2,102 participants revealed unprecedented knowledge deficits, with only 5.9% having heard of fatty liver disease and a mere 2.8% understanding its preventable nature. The finding that 94.0% of participants were “not sure” rather than definitively saying “no” about fatty liver awareness suggests a complete information void rather than misconceptions. This represents a far more severe awareness crisis than studies from Saudi Arabia, where less than half of the participants were unaware of MASLD, compared to our finding of 94% complete unawareness. Beyond confirming the low awareness of MASLD in India, our study contributes new insights by highlighting the strong knowledge-to-action relationship and a noteworthy pattern suggesting that higher formal education does not always translate into proactive liver health testing.

These findings add to existing international literature by identifying unique socio-cultural factors influencing liver testing behaviour in low- and middle-income country settings.

Our study findings strongly align with previous US data, revealing a widespread and concerning lack of awareness about MASLD. Alqahtani et al. (2021) [17] found that approximately 96% of adults with MASLD were unaware of their condition, closely matching our finding that 94% of participants were “not sure” about fatty liver disease. This significant knowledge gap highlights the urgent need for comprehensive health education initiatives to increase public awareness about NAFLD and its health consequences. The US study also identified young adults and ethnic minorities as notably lacking awareness patterns consistent with our findings, suggesting that targeted educational approaches may be necessary for these groups. The similarity of these results across different populations emphasizes the global need for programs to enhance understanding of MASLD and its complications, ultimately supporting early lifestyle interventions and reducing the future health burden of this disease.

Our comprehensive disease recognition analysis revealed that 90.1% of participants were unaware of any liver-associated conditions, with only 9.4% recognizing cancer, 8.4% hepatitis, and 5.8% fatty liver as liver diseases. Risk factor awareness was equally poor, with 95% uncertain about all fatty liver risk factors, and only 5% identifying excessive alcohol consumption as a risk. This limited recognition of alcohol as a risk factor for MASLD represents an incomplete understanding of the disease’s etiology. Knowledge of protective measures was also severely restricted, with only 9.4% recognizing a balanced diet and 8.9% identifying regular exercise as protective factors.

These observations align with the growing body of literature emphasizing the role of lifestyle factors and metabolic abnormalities in the pathogenesis of non-alcoholic fatty liver disease and its progression to hepatocellular carcinoma (Huang et al., 2021) [18]. The limited awareness observed in our study underscores the necessity for health education initiatives and lifestyle interventions at the population level. Such measures are crucial to reducing the future burden of fatty liver disease and its complications, particularly in the context of a rapidly growing obesity and diabetes epidemic.

The logistic regression analysis provided robust evidence for the knowledge-to-action pathway. Education emerged as the strongest predictor of liver testing behaviour, with graduates and above showing a 4.5% testing rate and 8.35-fold higher odds of being tested compared to non-graduates (OR = 8.35, 95% CI: 3.80–18.37). Knowledge score demonstrated a critical pathway, with participants having higher liver function knowledge (≥2 correct answers) showing dramatically elevated testing rates of 12.9% versus 1.2% for those with low knowledge (OR = 9.35, 95% CI: 4.2–20.8). Employment status also significantly predicted testing behaviour (OR = 5.42), suggesting workplace health benefits facilitate access to liver screening. Sensitivity analysis using an alternative classification of education (tertiary vs. non-tertiary) yielded results consistent with the primary analysis, although wider confidence intervals were observed due to small subgroup sizes.

Unlike countries such as Japan with universal mandatory workplace health screenings, India has a more limited regulatory framework. The Factories Act of 1948 mandates annual health checkups only for workers in manufacturing industries and hazardous processes, while the Occupational Safety, Health and Working Conditions (OSHWC) guidelines require periodic medical screenings primarily for employees in high-risk industries or those exposed to occupational hazards. Most service sector employees—who comprised 13.9% of our study population—are not covered by these mandatory screening requirements. Importantly, even where mandatory checkups exist under the Factories Act, these focus on occupational safety rather than comprehensive health screening, with specific tests varying by job profile and manufacturing process (e.g., hand examinations for food industry workers, chest X-rays for hazardous processes). Most corporate health checkups in India are voluntary employer-provided benefits rather than legal mandates, with studies showing that 82% of firms agree that annual health checkups increase productivity, but these remain optional initiatives. Therefore, the higher testing rates among employed individuals in our study (6.1% vs. 1.2% among unemployed, OR = 5.42, 95% CI: 2.8–10.5) likely reflect several factors beyond mandatory screening:

**1. Voluntary employer-provided health benefits**: Many companies offer annual health checkup packages as part of employee welfare initiatives, with liver function tests potentially included in comprehensive screening panels
**2. Better healthcare access and insurance coverage**: Employed individuals often have health insurance through employers, reducing financial barriers
**3. Workplace health awareness**: Corporate wellness initiatives and health camps conducted at workplace premises promote preventive healthcare culture
**4. Economic capacity**: Stable income enables discretionary healthcare spending for voluntary health assessments
**5. Social networks**: Workplace environments facilitate health information sharing and peer influence regarding preventive care

This Indian context differs markedly from countries with universal workplace screening mandates, making our employment-testing association more indicative of differential healthcare access and health-seeking behavior rather than policy-driven screening compliance. This distinction strengthens our argument for targeted interventions among the 63.8% unemployed population who lack both the indirect health benefits of employment-based wellness programs and the economic means for voluntary health screening.

Unemployment and lower levels of education have been linked to poorer access to health information and services that help prevent illness [19]. This represents a far more severe crisis than studies from Saudi Arabia, where less than half of the participants were unaware of MASLD, compared to our finding of 94% complete unawareness [20]. Only 1.6% of our respondents knew that liver tests are part of regular health check-ups, pointing to a weak connection between general health awareness and liver health. Our findings are supported by a study from Maharashtra that found people with higher education levels had better awareness of MASLD [21]. When it comes to knowledge, only 1.1% of participants knew that non-alcoholic beverages could lead to fatty liver, and merely 1.7% knew that it can be cured if caught early. Similar results were seen in a study from India that highlighted risky dietary habits like eating fried, spicy foods, and non-vegetarian items as contributors to MASLD [22]. Our comprehensive disease recognition analysis revealed that 90.1% of participants were unaware of any liver-associated conditions, with only 9.4% recognizing cancer, 8.4% hepatitis, and 5.8% fatty liver as liver diseases. Risk factor awareness was equally poor, with 95% uncertain about all fatty liver risk factors, and only 5% identifying excessive alcohol consumption as a risk.“ [21]. Our correlation analysis found a strong link between knowledge and awareness, especially regarding risk factors. This means that improving people’s knowledge will also increase awareness. This is backed by studies like one from Tincopa et al. (2021) [23], which found that while patients understood lifestyle changes were important, they didn’t fully grasp how NAFLD progresses. This is also supported by findings from previous awareness studies, which show that improved knowledge can translate into better health practices and screening uptake [24]. Similarly, a study by Jang et al. (2021) found that many patients lacked consistent information and needed better support from healthcare providers to manage the disease effectively [25].

Our demographic analysis revealed a concerning pattern, with 63.8% of participants unemployed despite 31.1% having a graduate degree or higher (27.4% undergraduates and 3.7% postgraduates), suggesting possible underemployment or limited job opportunities rather than a true employment–education paradox.

These factors matter because education and employment are linked to people’s knowledge of health and their access to healthcare. The high unemployment rate despite substantial educational attainment suggests potential economic challenges or employment barriers that may limit access to health information and services.

The findings of Giammarino et al. (2022) align with our observation that socioeconomic factors profoundly influence health outcomes, particularly in the context of nonalcoholic fatty liver disease. The study demonstrated that higher socioeconomic deprivation, measured by a composite of indicators such as education, employment, healthcare coverage, and income, was strongly associated with a greater likelihood of developing NASH and more severe fatty change in the liver [26].

Importantly, this association remained significant even after controlling for age, sex, race, s, and diabetes, suggesting that the effects of socioeconomic context operate independently of these health factors and reflect a true health disparity related to the conditions people live in. This underscores the pattern observed in our population, where higher education did not necessarily translate into lower disease risk when employment opportunities and other socioeconomic conditions were limited.

The Giammarino study highlights how barriers related to employment, healthcare access, and living conditions can undermine health outcomes, regardless of education level.

The study documented alarming patterns of healthcare underutilization. Only 1.6% of respondents knew that liver tests are part of regular health check-ups, pointing to a weak connection between general health awareness and liver health. Most concerning was that 84.7% of participants could not recall their last health check-up, with only 8.0% having had checkups within 6 months. This behavior pattern suggests poor health-seeking behavior, consistent with other Indian studies indicating that preventive health practices are generally low in the population [27]. Our study demonstrates a clear positive association between greater knowledge of liver health—including causes, symptoms, and preventive measures—and the likelihood of participants undergoing liver testing. This finding aligns with emerging evidence from Indian settings highlighting the critical role of awareness in fostering proactive health-seeking behaviors. A recent multicenter KAP survey conducted among Indian patients with metabolic disorders found that individuals with higher knowledge scores—reflecting better understanding of MASLD risk factors and complications—were significantly more likely to engage in preventive practices, including diagnostic testing and lifestyle modifications [28]. Therefore, our results—demonstrating that participants with higher liver health knowledge are more likely to undergo testing—are consistent with both patient- and provider-focused studies in India. These findings underscore the importance of educational interventions in promoting early detection and prevention of liver disease.

Our study’s significant gaps in knowledge and awareness highlighted the urgent need for comprehensive public health education in Jaipur, Rajasthan. Educational efforts are needed to focus on providing information about liver functions, the preventable nature of fatty liver disease (FLD), and the importance of regular health check-ups. Using demographically appropriate and easy-to-understand materials can help address these gaps. In addition, including liver health in routine medical checks improves awareness and allows early detection. A relevant example of effective educational intervention comes from a study conducted in Delhi NCR, where targeted programs significantly improved knowledge, attitudes, and practices related to Hepatitis B and C among school students. This study demonstrated that structured educational sessions could substantially enhance adolescents’ understanding and awareness of liver diseases [29]. Furthermore, the Government of India’s integration of Non-Alcoholic Fatty Liver Disease (NAFLD) into the National Programme for Prevention and Control of Non-Communicable Diseases (NP NCD) reflects a strategic approach to addressing liver health at the national level. This initiative emphasizes health promotion and early detection, aiming to ensure timely and appropriate care for individuals affected by MASLD [30].

The extremely low overall testing rate (37 out of 2,102 participants, 1.8%) indicates the population’s massive underutilization of liver health screening. The strong associations with education, employment, and knowledge scores suggest that liver testing is primarily accessed by socioeconomically advantaged individuals with better health literacy. The confluence of unemployment (63.8% of the sample) and low testing rates among this group represents a critical public health gap requiring targeted intervention.

The dramatic difference in testing rates between high-knowledge participants (≥2 correct liver function answers: 12.9%) and low-knowledge participants (<2 correct: 1.2%) demonstrates that educational interventions could substantially improve screening uptake. Education and knowledge emerged as the strongest predictors, suggesting that health literacy, rather than demographic factors, primarily drives liver testing behavior. These findings underscore the urgent need for comprehensive public health education targeting liver health awareness, particularly given India’s rising MASLD prevalence and the young demographic profile of our study population.

The small number of participants who had undergone liver testing (n = 37) limits the precision of odds ratio estimates, as reflected in wide confidence intervals. This was particularly evident in the knowledge score analysis, where the odds ratio of 48.7 had vast confidence intervals (2.2–1076.4), reflecting the rarity of high knowledge and testing behaviour in this population. Additionally, the cross-sectional design prevents the determination of causality between knowledge and testing behaviour.

To address the catastrophic MASLD awareness crisis documented in this study, immediate multi-level interventions are essential. Population-wide educational campaigns should target the 94% unaware of fatty liver disease, utilizing culturally appropriate materials in Hindi and English. Healthcare system integration must include mandatory liver health education for providers, routine screening protocols, and targeted programs for the 63.8% of the population who are unemployed and lack workplace health benefits. School-based education programs are crucial given the young demographic profile, while workplace wellness initiatives should leverage the 5.4-fold higher testing rates among employed individuals Policy interventions should strengthen NP NCD NAFLD (now MASLD) components, develop regional health strategies addressing the gap between educational attainment and employment opportunities, and establish public–private partnerships for broader program reach.

Long-term sustainability requires healthcare system capacity building, performance indicators linking facility funding to liver health metrics, and inter-sectoral collaboration with education, employment, and community organizations.

## References

[1] Younossi ZM, Golabi P, Paik JM, Henry A, Van Dongen C, Henry L. The global epidemiology of nonalcoholic fatty liver disease (NAFLD) and nonalcoholic steatohepatitis (NASH): a systematic review. Hepatology. 2023;77(4):1335–47. doi: 10.1097/HEP.0000000000000004 36626630 PMC10026948

[2] Misra A, Singhal N, Sivakumar B, Bhagat N, Jaiswal A, Khurana L. Nutrition transition in India: secular trends in dietary intake and their relationship to diet-related non-communicable diseases. J Diabetes. 2011;3(4):278–92. doi: 10.1111/j.1753-0407.2011.00139.x 21649865

[3] Vos T, Lim SS, Abbafati C, Abbas KM, Abbasi M, Abbasifard M, et al. Global burden of 369 diseases and injuries in 204 countries and territories, 1990–2019: a systematic analysis for the Global Burden of Disease Study 2019. The Lancet. 2020;396(10258):1204–22. 10.1016/S0140-6736(20)30925-9 PMC7567026 33069326

[4] Ekstedt M, Nasr P, Kechagias S. Natural history of NAFLD/NASH. Curr Hepatol Rep. 2017;16(4):391–7. 29984130 10.1007/s11901-017-0378-2 PMC6022523

[5] Bazick J, Donithan M, Neuschwander-Tetri BA, Kleiner D, Brunt EM, Wilson L, et al. Clinical Model for NASH and Advanced Fibrosis in Adult Patients With Diabetes and NAFLD: Guidelines for Referral in NAFLD. Diabetes Care. 2015;38(7):1347–55. doi: 10.2337/dc14-1239 25887357 PMC4477334

[6] Tevar AD, Clarke C, Wang J, Rudich SM, Woodle ES, Lentsch AB, et al. Clinical review of nonalcoholic steatohepatitis in liver surgery and transplantation. J Am Coll Surg. 2010;210(4):515–26. doi: 10.1016/j.jamcollsurg.2010.01.020 20347746 PMC4607044

[7] Asrani SK, Devarbhavi H, Eaton J, Kamath PS. Burden of liver diseases in the world. J Hepatol. 2019;70(1):151–71. doi: 10.1016/j.jhep.2018.09.014 30266282

[8] Rowe IA. Lessons from Epidemiology: The Burden of Liver Disease. Dig Dis. 2017;35(4):304–9. doi: 10.1159/000456580 28468017

[9] Das K, Das K, Mukherjee PS, Ghosh A, Ghosh S, Mridha AR, et al. Nonobese population in a developing country has a high prevalence of nonalcoholic fatty liver and significant liver disease. Hepatology. 2010;51(5):1593–602. doi: 10.1002/hep.23567 20222092

[10] Singh SP, Nayak S, Swain M, Rout N, Mallik RN, Agrawal O, et al. Prevalence of nonalcoholic fatty liver disease in coastal eastern India: a preliminary ultrasonographic survey. Trop Gastroenterol. 2004;25(2):76–9. 15471321

[11] Lazarus JV, Mark HE, Villota-Rivas M, Palayew A, Carrieri P, Colombo M, et al. The global NAFLD policy review and preparedness index: Are countries ready to address this silent public health challenge?. J Hepatol. 2022;76(4):771–80. doi: 10.1016/j.jhep.2021.10.025 34895743

[12] Hu G, Qiao Q, Tuomilehto J, Balkau B, Borch-Johnsen K, Pyorala K, et al. Prevalence of the metabolic syndrome and its relation to all-cause and cardiovascular mortality in nondiabetic European men and women. Arch Intern Med. 2004;164(10):1066–76. doi: 10.1001/archinte.164.10.1066 15159263

[13] Leung CM, Lai LSW, Wong WH, Chan KH, Luk YW, Lai JY, et al. Non-alcoholic fatty liver disease: an expanding problem with low levels of awareness in Hong Kong. J Gastroenterol Hepatol. 2009;24(11):1786–90. doi: 10.1111/j.1440-1746.2009.05914.x 19686415

[14] Duseja A, Chalasani N. Epidemiology and risk factors of nonalcoholic fatty liver disease (NAFLD). Hepatol Int. 2013;7 Suppl 2:755–64. doi: 10.1007/s12072-013-9480-x 26202291

[15] Cleveland ER, Ning H, Vos MB, Lewis CE, Rinella ME, Carr JJ, et al. Low Awareness of Nonalcoholic Fatty Liver Disease in a Population-Based Cohort Sample: the CARDIA Study. J Gen Intern Med. 2019;34(12):2772–8. doi: 10.1007/s11606-019-05340-9 31595464 PMC6854130

[16] Mondal R, Sarker RC, Acharya NP, Banik PC. Effectiveness of health education-based conventional intervention method to reduce noncommunicable diseases risk factors among rural population. Cardiovasc Diagn Ther. 2019;9(1):30–4. doi: 10.21037/cdt.2018.10.09 30881874 PMC6382656

[17] Alqahtani SA, Paik JM, Biswas R, Arshad T, Henry L, Younossi ZM. Poor Awareness of Liver Disease Among Adults With NAFLD in the United States. Hepatol Commun. 2021;5(11):1833–47. doi: 10.1002/hep4.1765 34558829 PMC8557315

[18] Huang DQ, El-Serag HB, Loomba R. Global epidemiology of NAFLD-related HCC: trends, predictions, risk factors and prevention. Nat Rev Gastroenterol Hepatol. 2021;18(4):223–38. doi: 10.1038/s41575-020-00381-6 33349658 PMC8016738

[19] Glass L, Asefa H, Volk M, Lok AS, Tincopa MA. Disease Knowledge, Health-Related Quality of Life, and Lifestyle Behavior Change in Patients with Nonalcoholic Fatty Liver Disease: Impact of an Educational Intervention. Dig Dis Sci. 2022;67(6):2123–33. doi: 10.1007/s10620-021-07052-9 34043121

[20] Abdulfattah AA, Elmakki EE, Maashi BI, Alfaifi BA, Almalki AS, Al Alhadi N, et al. Awareness of Non-alcoholic Fatty Liver Disease and Its Determinants in Jazan, Saudi Arabia: A Cross-Sectional Study. Cureus. 2024. 10.7759/cureus.53111 PMC10897741 38414702

[21] Lavekar A, Satyanarayana PT, Lavekar A. How is silent ‘non-alcoholic fatty liver disease’ epidemic perceived in India? A community based cross sectional study. Int J Community Med Public Health. 2019;6(12):5256. doi: 10.18203/2394-6040.ijcmph20195481

[22] Singh SP, Singh A, Misra D, Misra B, Pati GK, Panigrahi MK, et al. Risk Factors Associated With Non-Alcoholic Fatty Liver Disease in Indians: A Case-Control Study. J Clin Exp Hepatol. 2015;5(4):295–302. doi: 10.1016/j.jceh.2015.09.001 26900270 PMC4723647

[23] Tincopa MA, Wong J, Fetters M, Lok AS. Patient disease knowledge, attitudes and behaviours related to non-alcoholic fatty liver disease: a qualitative study. BMJ Open Gastroenterol. 2021;8(1):e000634. doi: 10.1136/bmjgast-2021-000634 34193468 PMC8246278

[24] Tan C-K, Goh GB-B, Youn J, Yu JC-K, Singh S. Public awareness and knowledge of liver health and diseases in Singapore. J Gastroenterol Hepatol. 2021;36(8):2292–302. doi: 10.1111/jgh.15496 33735936 PMC9290627

[25] Jang Y, Lee JY, Kim SU, Kim B. A qualitative study of self-management experiences in people with non-alcoholic fatty liver disease. Nurs Open. 2021;8(6):3135–42. doi: 10.1002/nop2.1025 34405583 PMC8510709

[26] Giammarino AM, Qiu H, Bulsara K, Khan S, Jiang Y, Da BL. Community socioeconomic deprivation predicts nonalcoholic steatohepatitis. Hepatol Commun. 2022;6(3):550–60. 34668658 10.1002/hep4.1831 PMC8870017

[27] Yadav R, Zaman K, Mishra A, Reddy MM, Shankar P, Yadav P. Health Seeking Behaviour and Healthcare Utilization in a Rural Cohort of North India. Healthcare. 2022;10(5):757. doi: 10.3390/healthcare10050757 35627894 PMC9140543

[28] Shaikh S, Negalur V, Patil S, Dargad R, Jaiswal A, Bhandari A, et al. Knowledge-Attitude-Practices of Non-Alcoholic Fatty Liver Disease among Indian Patients with metabolic disorders: A Multi-centric Cross-Sectional Observational Study. Int J Med Sci Clin Invent. 2024;11(10):7260–7.

[29] Kaushal K, Aggarwal P, Dahiya N, Kumar G. Impact of educational interventions on hepatitis B and C awareness among school students of Delhi NCR, India. BMC Public Health. 2024;24(1):2112. doi: 10.1186/s12889-024-19577-5 39103833 PMC11299276

[30] Venugopal V, Richa R, Singh D, Gautam A, Jahnavi G. National Programme for Prevention and Control of Cancer, Diabetes, Cardiovascular Diseases, and Stroke: A Scoping Review in the Context of Hypertension Prevention and Control in India. Indian J Public Health. 2023;67(Suppl 1):S50–7. doi: 10.4103/ijph.ijph_681_23 38934882

